# Inflammatory bowel disease associated knowledge in South Indian populations: rational Study

**DOI:** 10.4314/ahs.v23i2.51

**Published:** 2023-06

**Authors:** Sathyaprabha Gopalan, Srinivasan Nagarajan, Aravindh Somasundaram, Manisenthilkumar Kulanthaivelu Thiyagarajan

**Affiliations:** 1 Department of Pharmacy, Annamalai University, Chidambaram, (Tamil Nadu) India; 2 Department of Pharmacy Practice, KMCH College of Pharmacy (Affiliated to TN. Dr.M. G. R. Medical University), Coimbatore, (Tamil Nadu) India; 3 Department of Gastroenterology, Kovai Medical Center and Hospital, Coimbatore, (Tamil Nadu) India; 4 Department of Pharmacy and Quality Control, Royal Care Super Specialty Hospital, Coimbatore, (Tamil Nadu), India

**Keywords:** Inflammatory bowel disease, South Indian populations

## Abstract

**Background:**

**Objectives:**

The pivotal of the study was to compare the effectiveness of education in disease-associated knowledge of Inflammatory Bowel Disease (IBD) patients between pre-test and post-test using the IBD-KNOW questionnaire and patient educational resources.

**Methods:**

This study used a patient proforma and IBD- KNOW questionnaire to perform the study prospectively by interviewing method. The patient selection was based on inclusion and exclusion criteria by convenient sampling technique from November 2018 to July 2019 at a multispecialty hospital. Knowledge scores and inter-item correlation were calculated between the pre-test and post-test by R Programming software with p<0.05.

**Results:**

Among 40 patients with IBD diagnosis, the baseline sociodemographic characteristics were recorded. The response rate of IBD knowledge between the pre-test and post-test resulted in significant differences with varying scales but the response rate was lesser in the domains of management and pregnancy-based questions in the pre-test and post-test.

**Conclusions:**

Recently there was a swift in IBD incidence, this may be improved by affording suitable patient education and counseling for further knowledge level in managing the disease by coping strategy. On comparison between the pre-test and post-test, this study recommends innovative educational methods to enable continuing education for chronic disease which can be easily accessible and reliable for IBD patients.

## Introduction

An Inflammatory Bowel Disease (IBD) is a chronic gastrointestinal disorder caused by the dysregulated immune response in the gut of the host intestinal microflora. IBD is comprised of idiopathic intestinal disease, characterized by its location with the involvement of disease in the bowel wall^1^. Two types of IBD, ulcerative colitis (UC) and Crohn's disease (CD) are seen in which diffused inflammation of the colonic mucosa occurs in ulcerative colitis (UC)^2^ whereas transmural ulceration on any portion of the gastrointestinal tract (GI) results in Crohn's disease (CD) 3. Crohn's disease can affect any region of the intestine in a discontinuous pattern involving the terminal ileum, cecum, perianal area, and colon. In contradiction, ulcerative colitis can affect part of the colon or the entire colon and the rectum in a continuous pattern^4^. A thickened submucosa, transmural inflammation, fissuring ulceration, and granulomas were exhibited histologically in Crohn's disease, whereas in ulcerative colitis the inflammation is limited to the mucosa, submucosa with cryptitis and crypt abscesses 5.

The prevalence rate of IBD becomes rapid in developing countries with the changing view as the disease of the western population in one-twenty million population. In India, the total case of IBD was the largest with a rate of 1.4 million population in 2010 next to the USA around 1.64 million cases^6^. Crohn's disease was more usual in South India than ulcerative colitis in North India. Population-based studies in India were very few, however, there is a greater number of IBD cases in younger patients with disease severity in their clinics and hospitals 7. Epidemiological studies have been hindered by various factors like appropriate databases, registries, scarcity of healthcare and hospital-based population studies proposed the highest disease burden in Asian countries 8.

Barriers in the management and IBD care of the patients have not been previously described by the pharmacist, however, there was no data on concerns with exposure and IBD knowledge^9^. Deficits in the identification of knowledge evaluation play a major role in the effect of disease management. Knowledge acquisition was an integral role in the diagnosis and treatment of self-management skills of the disease 10. Earlier studies by western countries explained the gaps in the level of knowledge specific to IBD complications and various treatment options. So routine assessment will be a requisite for chronic conditions of diseases like IBD 11. Educational resources and health care facilities for individual factors play a role in patient education and the interventional effect on patient outcomes. There were no parameters in the assessment of specific standards but intervention may substantially change the disease concern by providing structural information to the study population 12.

## Aim

The purpose of the study was to evaluate the sociodemographic characteristics and the effectiveness of patient education in the pre-test and post-test of the prospective study design by measuring the level of knowledge in IBD patients. The hypothesis of the research would demonstrate that patient education may improve the knowledge scores in the post-test of the study.

## Methods

### Ethics Approval

The Institutional Human Ethics Committee (IHEC) approved for ethical clearance before the commencement of the study and the proposal number was EC/AP/591/10/2018. Participation of the patient in the study was enrolled after the informed written consent through the direct interview technique.

### Study Design and Participants

A prospective interventional study was conducted at Kovai Medical Center and Hospital (KMCH) in the Department of Gastroenterology from November 2018 to July 2019 by a convenient sampling technique. A total of 40 patients were enrolled in the study based on their inclusion and exclusion criteria. Inclusion criteria were patients with a diagnosis of IBD, adult population above eighteen years, and patient attentiveness during the study phase. Patients with intermediate colitis, irritable bowel syndrome, diagnosis of colorectal cancer, and severe illness patients were excluded from the study.

### Data Collection Tool

The performa was specially designed that comprised of the closed-ended questionnaire with sociodemographic characteristics such as age, gnder, marital status, area of residence, social behavior (alcohol & smoking), and family history.

A validated IBD-KNOW questionnaire was used after obtaining permission from the author to measure the knowledge scores of the participants (Hyuk Yoon et al 2017). The Cronbach-alpha value of IBD-KNOW questionnaire α = 0.952. It is the first tool to be used in the South Indian population, as no previous studies were performed on disease-related knowledge.

By application of IBD-KNOW, predictive factors in disease related to knowledge levels can be evaluated. The questionnaire comprised of 24 item questions and scoring was calculated as one point for each correct answer (Yes =1 No=0 & Don't Know=0). The knowledge score was measured by a ratio as the correct answer rate for each question.

A patient information leaflet was developed logically and a readability assessment was done with the Flesch-Kincaid reading score used for the educational session of the patients during the visit. A pilot study was performed on a small population to examine the difficulty of respondents in interpreting the questionnaire.

### Data Collection Procedure

The patients were enrolled by the convenient method of sampling (n=40) based on inclusion and exclusion criteria. In the baseline visit (V0), the patient's data was collected using a proforma and IBD-KNOW questionnaire. Patient educational resources were provided to the participants with standard care during first visit (V1). After 12 weeks, patients who attended the second visit (V2 ) for physician review were assessed for IBD-KNOW in the post-test. The data were analyzed by R program and interpreted accordingly.

### Data analysis

Statistical analysis of the variables was conducted using the R program (Statistical tool for data analysis). The inter item correlation was calculated with the response rate of each question in both the pre-test and post-test using the Pearson correlation test. The chi-square test and independent student t-test were used for the estimation of study participants. The knowledge response rate was calculated as mean in percentage (%) in the study population and categorized as good for a maximum rate of above 70 %, average knowledge response of 35% to 70%, and a poor rate of knowledge response below 35%.

## Results

### Sociodemographic features in IBD patients

Among the 40 study population, the majority of patients were between 30 - 40 years 47.5%, with a female predominance of 62.5% than the male patients, marital status of a married person with 62.5%, Urban was greater than rural with 72.5%, Business patients was greater 27.5% with the disease, family history in 10% among the study population, among education status high school level with 47.5%, the social behavior of alcoholism in 15% and smoking habits with 17.5% as given in table 1.

**Table 1:** Frequency distribution of Sociodemographic features

Characteristics	Frequency (n=40)	Percentage (%)	Chi Square test	*p value
**Age in Years**				
20-30	7	17.5%	9.3	0.323
30-40	19	47.5%		
40-50	12	30%		
50-60	1	2.5%		
Greater than 60	1	2.5%		
**Gender**				
Male	15	37.5%	12.43	**0.007**
Female	25	62.5%		
**Marital Status**				
Married	25	62.5%	0.86	0.995
Unmarried	11	27.5%		
Divorce	1	2.5%		
Widow	3	7.5%		
**Area of Residence**				
Urban	29	72.5%	1.28	0.532
Rural	11	27.5%		
**Occupation**				
Professional	7	17.5%	32.77	**0.001**
Student	3	7.5%		
Home Maker	9	22.5%		
Business Agriculture	11 4	27.5% 10%		
Others	6	15%		
**Educational Status**				
Illiterate	4	10%		
Elementary	5	12.5%		**0.013**
High School	19	47.5%		
Graduate	12	30%		
**Family History**				
Yes	4	10%	5.38	0.076
No	36	90%		
**Alcoholism**				
Yes	6	15%	5.38	0.254
No	32	80%		
Past	2	5%		
**Smoking**				
Yes	7	17.5%	23.96	**0.001**
No	27	67.5%		
Past	6	15%		

* Note: p< 0.05 significance p<0.00 strongly significance

### Knowledge assessment in IBD patients

In the pre-test, the mean correct answer rate for 24 items in the question was 40.7%. The questions related to the treatment in the use of biological agents “Q17”, usage of enema or suppository in UC “Q18”, and in women-related questions “Q23” the rate of the correct answer was at least 12.5%. The highest rate of response was identified in “Q9” for the development of anemia after severe inflammation and in “Q14” the persistence of bowel inflammation even after treatment initiation was 72.5%. But the minimum response of < 35% existed in most of the questions related to anatomy 32.5%, function 27.5%, the occurrence of disease 30%, management 12.5%, long term effect and surgery 30%. (Table 2)

**Table 2:** Response rate in Knowledge score between Pre-test and Post-test

Knowledge Response Rate	Pre-test (n=40)	Post-test (n=37)
Maximum Rate >70%	Q9 Risk factors Q14 Symptoms	Q2Anatomy Q5 Diet Q8& Q10 Disease Occurrence Q9 Risk factors Q13& Q14 Symptoms
Average Rate (35 %- 70%)	Q2 Anatomy Q3 Function Q5 Diet Q7 Risk factors Q8& 10 Disease Occurrence Q13 Symptoms Q15&16 Management Q21 Long term effects & surgery	Q1 Anatomy Q3& 4 Function Q6 Smoking Q7 Risk factors Q11& 12 Disease Occurrences Q15&16 Management Q19, 20 & 21 Long term effects & surgery Q24 Vaccination
Minimum Rate < 35%	Q1 Anatomy Q4 Function Q6 Smoking Q11& 12 Disease occurrences Q17&18 Management Q19& 20 Long term effects & surgery Q22 &23 Pregnancy Q24 Vaccination	Q17 & 18 Management Q22 & 23 Pregnancy

**Fig 1:**
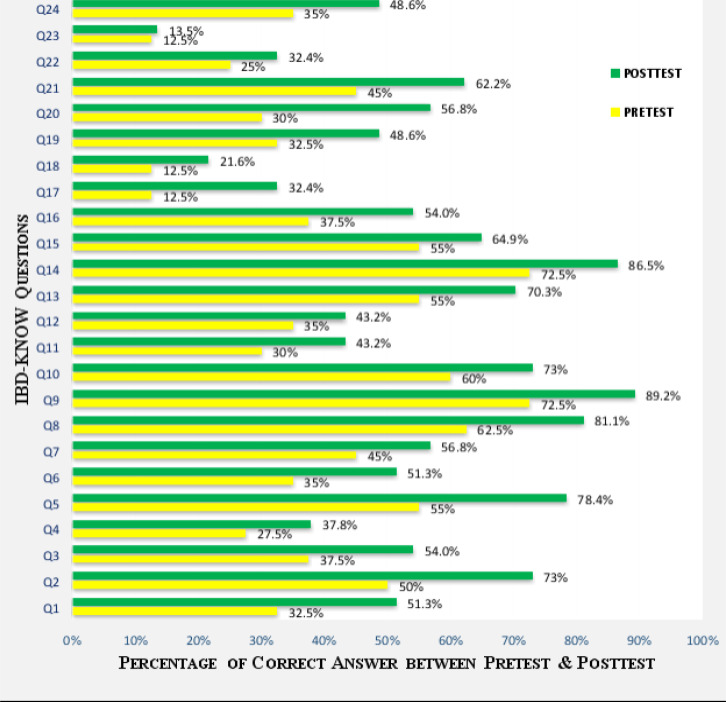
Distribution of response rate in domains of IBD knowledge questions

The patient education conducted through face-to-face interviews of enrolled patients was educated with a patient information leaflet for five minutes in the baseline study of visit 1 regarding the type of disease (occurrence, anatomy, function), risk factors, complication, social behavior, lifestyle modifications and management of the disease. On the second visit (V2 ) after 12 weeks, the patient enrollment was biased from ( n = 40 ) to ( n = 37 ) in the post-test of the study. Bias may be due to irregularity in the patient review with the physician or lack of time due to their work. The mean rate of the correct answer in the post-test was 53.37%. The lowest answer rate was in “ Q23 ” women-related questions at as13.5% and the highest rate was in “ Q9 ” for the development of anemia after severe inflammation at 89.2%. The maximum response was ascertained in “ Q2 ” anatomy as 72.9%, “ Q5 ” diet as 78.4%, “ Q8 ” as 81.1% & “ Q10 ” as 72.9% disease occurrence, “ Q13 ” & “ Q14 ” Symptoms 86.5% (Figure 1).

There exists a significant difference in response rate between the pre-test and post-test in the educational group of the patients by acceptance of an alternative hypothesis. The degree of correlation between the variables was within the limits of -0.1 to 0.1. (Table 3)

**Table 3:** Description of correct answer rate between pretest and post-test of the study

Domains in IBD KNOW Questionnaire	Pre- test n=40 (%)	Item Total Correlation	Post- test n=37 (%)	Item Total Correlation
Q1.The terminal ileum is the last part of the small bowel located in right lower abdomen.	13(32.5%)	0.831	19(51.4%)	0.805
Q2. The rectum is part of the colon starts approximately 15cm from the anus and finishes at the anus	20 (50%)	0.818	27(72.9%)	0.724
Q3 .The function of the colon is to absorb nutrients	15 (37.5%)	0.801	20(54.1%)	0.734
Q4. People can survive without the colon, but not without the small bowel	11 (27.5%)	0.803	14(37.8%)	0.828
Q5. Specific foods to be avoided in IBD are well known	22 (55%)	0.333	29(78.4%)	0.671
Q6. Smoking cessation is important to prevent worsening of Crohns disease	14 (35%)	0.729	19(51.3%)	0.892
Q7. Risk of IBD increases with family history of the condition	18 (45%)	-0.187	21(56.8%)	0.87
Q8. IBD can develop in all age groups, but is more frequent at younger ages	25 (62.5%)	0.325	30(81.1%)	0.589
Q9. Anemia may develop if severe inflammation persists	29 (72.5%)	0.571	33(89.2%)	0.766
Q10. Crohns disease can occur anywhere in the digestive tract, from the mouth to the anus	24 (60%)	0.256	27(72.9%)	0.681
Q11. Ulcerative colitis rarely involves the rectum	12 (30%)	-0.166	16(43.2%)	0.809
Q12. IBD can involve organs other than the bowels.	14 (35%)	0.823	16(43.2%)	0.879
Q13.IBD is considered cure if symptoms do not recur after a few years.	22 (55%)	0.833	26(70.3%)	0.972
Q14.Inflammation in the bowels may persist even if the symptoms improve after treatment initiation.	29 (72.5%)	0.696	32(86.5%)	0.766
Q15.Long term steroid administration is advised to reduce inflammation recurrence.	22 (55%)	0.833	24(64.9%)	0.839
Q16. Constant blood monitoring is indicated for patients who are on immunocompromised agents such as Azathioprine, because their WBC may decrease.	15 (37.5%)	0.801	20(54.1%)	0.934
Q17.Biological agents are mainly used in patients with mild symptoms.	5 (12.5%)	0.516	12(32.4%)	0.769
Q18. Suppository or enema is used to treat cecal inflammation in patients with Ulcerative Colitis.	5 (12.5%)	0.308	8(21.6%)	0.557
Q19. Patients with IBD for 8–10 yrs should have colorectal screening.	13 (32.5%)	0.478	18(48.6%)	0.848
Q20. Permanent colostomy is performed if surgery is indicated for patients with Ulcerative colitis.	12 (30%)	0.491	21(56.8%)	0.649
Q21. Patients with Crohns disease of the small bowel may be cured after surgery.	18 (45%)	0.51	23(62.2%)	0.835
Q22. Patients with IBD should stop all the medications when considering pregnancy.	10 (25%)	0.756	12(32.4%)	0.737
Q23. Most patients with IBD are advised cesarean section delivery.	5 (12.5%)	0.411	5(13.5%)	0.479
Q24. Immunocompromised patients with IBD should avoid any kind of vaccination	14 (35%)	0.686	18(48.6%)	0.707

## Discussion

This was the first study in south India on health-related knowledge determination which forms the base for the educational program and further follow-up in IBD patients. The age factor was reliable to the study that disease was more prevalent in the mean age of 33.5 ± 13.1 years with the greater years of age between 30 to 40 years^13^. The predominance of females over males was similar to the earlier studies 14. Family history showed similarities to north India than the developed countries the prevalence among the first relative was greater in siblings than the parents with a higher probability 15. The results of various studies described the relation between IBD and alcoholic habits as they do not know the frequency and quantity of alcohol intake by IBD patients and resembled the present study 16. The area of the residence (urban and rural) as there was a positive association of IBD in urban people due to lifestyle which may exacerbate or induce the IBD condition 17.

The present study on knowledge assessment was based on IBD -KNOW questionnaire, which was exempted from other studies related to CCKNOW (Crohn's Colitis Knowledge questionnaire) which had few problems in the clinical evaluation of IBD knowledge. However, the usage of biologicals in IBD treatment was the updated item in the IBD KNOW questionnaire but the question “ Q17 ” about biologicals in the management of mild symptoms was less in the pre-test compared to the post-test^18^. The quality of care by avoiding vaccination in immunocompromised patients “ Q24 ” was relatively effective in the management of IBD 19. Similarly, the present study explored a few variations than the pretest of 35% with a low response rate. In IBD women patient's knowledge regarding the plan for pregnancy to stop the medication in “ Q22 ” was the lowest in both pre-test and post-test similar to the study stating that females were less than male patients as they may face problems during pregnancy 20. The questions related to the complication were greater in IBD patients and this was reliable to the previous study 21. The correct answer rate in the drug management was lesser than 50% both in pre-test as well as post-test mentions for reliability in older studies^22^. The improvement in knowledge score in the post-test was reliable to the study stating that formal education supports the IBD population like other chronic diseases and higher knowledge levels had the least worries and concerns about the disease. The present study was not consistent with the past study with reduced health care on long-term educational benefits 23. The outcome will be more effective when education on the management with drugs were provided by proper training in the IBD patients was similar to the present study 24. The appropriate review, consistent follow-up, and drug compliance were the significant features in the management of IBD for lowered relapse in the frequency of disease 25.

## Conclusion

The study revealed the effectiveness of education in post-test by greater than 50% in disease associate knowledge. The humanistic approach by pharmacists aids the patients to motivate and support in reducing the disease severity. The present study recommends affording suitable education to improve knowledge levels that help to manage the disease and improves the coping strategy. Recently there was a swift in IBD incidence, but the progress of effective educational programs on management for IBD was still scarce in India. Effective educational modalities for disease-related knowledge can be provided by telemonitoring, web-based teaching, and tele-education. Also training the health care professionals may lessen the disease burden in IBD patients. As a consequence, of the study, there is a need for the development of innovative educational methods to enable continuing education for chronic disease which can be easily accessible and reliable for IBD patients.
